# Effects of Iron Supplementation on Erythropoietic Response in Patients with Cancer-Associated Anemia Treated by Means of Erythropoietic Stimulating Agents

**DOI:** 10.5402/2011/108397

**Published:** 2011-10-13

**Authors:** Torbjörn Karlsson

**Affiliations:** Department of Hematology, Uppsala University Hospital, 751 85 Uppsala, Sweden

## Abstract

During the past decade, intravenous iron supplementation to ESA (erythropoiesis-stimulating agent) therapy has emerged as an option to augment hemoglobin response in anemic cancer patients. In this paper, the results of seven published randomized clinical trials assessing the role of iron supplementation to ESA therapy in the hematology/oncology setting will be discussed. The pathogenetic mechanisms behind functional iron deficiency, a major reason for ESA hyporesponsiveness in cancer, will also be described.

## 1. Introduction

Recombinant human erythropoietin (rHuEPO) has revolutionized the treatment of anemia associated with chronic kidney disease (CKD). A few years after the publication of the trial on rHuEPO treatment in the nephrology setting by Eschbach and coworkers [1], reports on ESA treatment of anemia associated with solid tumors as well as hematologic malignancies were published [2–4]. Although epoetin and darbepoetin alfa treatment of such patients increases hemoglobin, reduces transfusion requirements, relieves symptoms of anemia, and improves health-related quality of life [5–8], the efficacy is less than that in the nephrology setting with hemoglobin responses (defined as an increase in Hb > 20 g/L, or Hb increased to 120 g/L or more) typically about 60% [6, 9–11].

A major reason why anemic cancer patients do not respond to ESAs is functional iron deficiency (FID), that is, failure to provide erythropoiesis with iron despite sufficient iron stores. The mechanism behind FID has been elucidated in recent years [12, 13]. An inflammatory response induced by conditions such as cancer increases the expression of the hepatic protein hepcidin in an interleukin-6-dependent manner [14]. Secreted hepcidin binds to the transmembrane iron-transporting protein ferroportin in macrophages of the reticuloendothelial system (RES) as well as in hepatocytes and in enterocytes and downregulates its expression by posttranslational mechanisms [15]. In addition to decreased transport of iron from enterocytes to the circulation, the downregulated ferroportin expression reduces iron efflux from macrophages [16, 17], thus making less iron available for erythropoiesis, the ultimate consequence of which is FID and anemia [18]. FID has also been observed in CKD patients treated with ESAs [19]. This variant of FID can occur without any inflammatory response with iron blockage in macrophages and hepatocytes, but the underlying mechanism is an ESA-driven supraphysiological erythropoiesis in which the rate-limiting step is the delivery of iron from its stores to erythroblasts. One way to avoid the relative iron deficiency in FID is the addition of intravenous (i.v.) iron to ESA therapy, which has been found effective in the nephrology setting [19]. It has also been shown that i.v. iron alone raises Hb in anemic CKD patients with FID [20]. 

 For noninvasive diagnosis of FID, the combination of more than 100 *μ*g/L ferritin and less than 20% transferrin saturation (TSAT) has been proposed [19, 21]. Although the diagnosis of absolute or functional iron deficiency is straightforward to diagnose in otherwise healthy individuals, it is often more difficult to diagnose in patients who suffer from cancer, infections, or inflammatory diseases since the biochemical iron status is affected by acute-phase responses [22]. Under these circumstances, the best way to assess the iron status of an individual is to perform a bone marrow iron staining [23]. Thus, in the hematology setting where bone marrow examination is routinely performed, the optimal definition of FID is probably a positive bone marrow iron staining in combination with TSAT < 20%. 

The current American and European hematology/oncology guidelines recommend i.v. iron supplementation to cancer patients on ESA therapy with iron deficiency [24] and absolute or functional iron deficiency [25], respectively, but do not consider i.v. iron supplementation as a care standard in this setting. In this paper, I will focus on the seven published randomized clinical trials (RTCs) assessing the role of iron supplementation in cancer patients on ESA therapy [26–32].

## 2. Characterization of Study Subjects, ESA, and Iron Doses as well as Preparations

These seven RCTs were published between 2004 and 2011 and include a total of 1703 patients, the largest reporting the results for 502 and the smallest for 67 patients [28, 32]. The type of ESA used was darbepoetin in four of the trials and Epo alfa or Epo beta in the other three. No increase in ESA dose was allowed in the trials by Auerbach, Bastit and Steensma, whereas ESA dose escalation was permitted in the other three trials if no Hb increment of at least 1 g/dL was seen after 4 weeks of treatment [27, 28, 30]. ESA dose reduction was allowed in all but one of the trials [26]. Six of the trials excluded patients with absolute iron deficiency, but only the Hedenus trial used positive bone marrow iron staining as an inclusion criterion [28]. The other 5 trials used different cut-off points for ferritin and TSAT to exclude patients with an absolute iron deficiency (Table 1). The Auerbach trial in 2004 probably included patients with an absolute iron deficiency since one inclusion criterion was ferritin ≤200 *μ*g/L or <300 *μ*g/L with transferrin saturation ≤19% [26].

Ferric gluconate was used as iron preparation in 4 of the trials, iron dextran in 2, and iron sucrose in one. The cumulative dose of intravenous iron in the trials differed at least four-fold, from 750 mg per patient (protocol) in the Pedrazzoli trial [30] to 3000 mg (administered) in the 2004 trial by Auerbach [26]. Iron was withheld in 4 of the studies if there were biochemical signs of iron overload [28–31]. The trial by Hedenus and coworkers [28] included patients with lymphoproliferative malignancies not receiving chemotherapy only, whereas the other six trials involved anemic patients with solid malignancies, the most common being breast, gastrointestinal, and lung cancers, as well as patients with hematologic malignancies on concomitant chemotherapy.

## 3. Summary of Results

In six of the RCTs discussed here, there was a significantly greater increase in Hb and/or a greater proportion of patients who achieved a hemoglobin response (defined as an increase in Hb > 20 g/L or Hb increase to 120 g/L or more or in the 2010 Auerbach trial target Hb ≥ 110 g/L) in ESA plus i.v. iron-treated patients (experimental population) compared to those treated with ESA plus oral iron or no iron (control population). Among the six trials with positive results, the increase in Hb varied from 19 to 29 g/L in the experimental populations compared to 9 to 16 g/L in the controls [26–31], whereas, in the Steensma trial, the increment in Hb was 26 and 24 g/L in the experimental and the control populations, respectively, with no significant difference between them [32]. The hemoglobin response in the six positive trials varied from 68 to 93 percent in the experimental populations compared to 25 to 73 percent in controls. In five of the trials, the time to achieve a hemoglobin response was significantly shorter in the experimental populations [26, 28–31]. In the Steensma trial, the hemoglobin responses in the experimental and control populations were 70 and 65%, respectively, a nonsignificant difference. Two of the trials reported that a significant greater proportion of patients with FID (TSAT < 20%) receiving intravenous iron supplementation achieved a hemoglobin response compared to those with TSAT > 20% [27, 28]. In all trials reporting EOTP (end of treatment period) ferritin or change in ferritin during treatment, ferritin decreased or increased less in controls compared to the experimental populations (Table 2). Absence of intravenous iron supplementation led to decreased EOTP TSAT compared to baseline in 3 of these trials, whereas this was not observed in the Steensma trial [32]. The Hedenus and Steensma trials assessed a possible ESA saving effect of iron supplementation, although cumulative ESA doses per patient did not differ significantly between the experimental and the control populations [28, 32]. The fraction of patients receiving transfusions with red blood cells varied from 1 to 25% in the trials, with one of them showing a significantly reduced need for transfusions in the experimental population compared to control [29]. Quality of life (QoL) was assessed in four of the trials, but no significant positive effects could be detected when comparing the experimental and the control populations [26, 29, 31, 32], whereas there was a positive correlation between Hb increment and positive effects on QoL in the trials by Auerbach and Steensma irrespective of treatment group [26, 32]. A suspected higher incidence of severe adverse events in the i.v. iron population in the Steensma trial [32] led to its premature termination, whereas acute adverse events including fatigue, myalgia, and nausea reported in the other trials were mild, except for 2 cases of iron-related anaphylactic reactions in the Auerbach 2010 trial [31].

## 4. Discussion

Although it has been known for many years that i.v. iron supplementation to ESA therapy can augment hemoglobin response in the nephrology setting [33, 34], the first trial showing an increased hemoglobin response due to iron supplementation in anemic cancer patients on ESA therapy was not published until 2004 [26]. The results of that trial have been confirmed by five other RCTs [27–31]. Previous trials including anemic cancer patients treated with ESAs have shown a typical hemoglobin response of about 60% [6, 9–11], a figure that increased significantly to 68–93% when ESA was supplemented with i.v. iron [26–31]. In contrast to the other six trials, the results of the Steensma trial [32] revealed no improvement in hemoglobin response after addition of i.v. iron to ESA therapy. There are several possible explanations for this lack of effect, two of which will be discussed here. Firstly, the Steensma trial included subjects with a TSAT of up to 60%. This inclusion criterion meant that patients with neither absolute nor functional iron deficiency, that is, patients less likely to benefit from iron supplementation, could be included. Furthermore, the dose of iron in the Steensma trial might have been too low to be efficient. The planned total dose of iron in this trial was the second lowest among the seven published trials, and the mean actual dose administered was the lowest [35]. In two of the trials, the hemoglobin response in the control populations was lower than expected, 25 and 41%, respectively, probably due to the shorter duration of these trials [26, 27] and that patients with absolute iron deficiency were not excluded [26]. The lowest efficient iron dose in these trials was 750 mg administered over 6 weeks [30]. 

Although there are many hematological and non-hematological benefits of iron [36], there are also safety concerns about iron supplementation. The most feared acute side effect of intravenous iron administration is the risk of anaphylactic reactions associated with iron dextran preparations. However, in the nephrology setting, the risk of acute severe adverse reactions after iron dextran administration is less than 1% [37]. The risk of anaphylaxis is even smaller with modern iron preparations [38, 39]. In agreement with this, the reported incidence of acute severe adverse reactions associated with intravenous iron in the RCTs discussed here was well below 1%. 

Two of the major concerns about long-term intravenous iron supplementation are the increased risks of bacterial infections and vascular damage induced by oxidative stress. It is known from the pre-rHuEPO era that heavily transfused CKD patients with biochemical signs of iron overload were at higher risk of bacterial infections [40, 41]. However, the epidemiological survey EPIBACDIAL performed after the advent of rHuEPO treatment could not identify elevated serum ferritin levels as a risk factor for bacterial infections in CKD patients [42]. The risk of iron overload after multiple iron infusions may be prevented by repeated measurements of ferritin and suspension from the infusions if biochemical signs of iron accumulation are observed [19]. The other major concern is that iron supplementation may accelerate atherosclerosis by oxidative stress, thereby increasing the risk of renal and cardiovascular morbidity [43, 44]. Intravenous iron administration in CKD patients leads to a rapid increase of nontransferrin bound redox-active iron and induces oxidative stress measured as an increase in lipid peroxidation [45, 46]. Since the oxidative stress induced by the different commercially available i.v. iron preparations does not seem to be of similar magnitude [47], it may be possible to reduce this risk by choosing one of the more stable ones. 

At least in theory, iron administration may decrease iron mobilization from its stores in FID, since diferric holotransferrin but not apotransferrin stimulates hepcidin expression [48]. However, a possible iron-induced hepcidin-mediated increased sequestration of iron in the RES must be disregarded due to its positive effects in FID since i.v. administered iron actually stimulates erythropoiesis in this condition. In absolute iron deficiency, i.v. administrated iron-carbohydrates rapidly enter RES macrophages, where they are processed, thus releasing iron from these cells and delivering it to erythroblasts by transferrin. In cancer patients, less iron may be delivered from the RES macrophages to erythroblasts [49, 50]. Since a fraction of iron in the iron-carbohydrate complex directly binds transferrin [51], it is possible that this fraction, which escapes sequestration in RES, mediates its biological effect in FID.

## 5. Concluding Remarks

Six of the seven RCTs discussed here show an augmented erythropoietic response in anemic ESA-treated cancer patients receiving i.v. iron supplementation compared to those not receiving iron. This effect was observed in patients with absolute iron deficiency, those who fulfilled the FID criteria as well as in those who did not. 

Intravenous iron supplementation should be strongly considered in anemic cancer patients treated with ESAs, since its positive effect on hemoglobin response has been documented in six published RCTs [26–31], two RCTs in abstract form [52, 53], and two meta-analyses [54, 55]. However, the preferred iron preparation, optimal dose of iron, and dosing schedule are currently not known.

## Figures and Tables

**Table 1:** Eligibility criteria (Hb, ferritin, TSAT, bone marrow iron staining) for the seven trials.

Trial	Hb (g/L)	Ferritin (*μ*g/L)	TSAT (%)	BM iron
Auerbach 2004 [26]	≤105	≤200	≤19*	ND
Auerbach 2010 [31]	≤100	≥10	≥15	ND
Bastit et al. [29]	<110	10–800	≥15	ND
Hedenus et al. [28]	90–110	<800	—	+
Henry et al. [27]	<110	100–900	15–35	ND
Pedrazzoli et al. [30]	<110	100–800	20–40	ND
Steensma et al. [32]	<110	≥20	<60	ND

*in combination with ferritin <300 *μ*g/L. Abbreviations: TSAT, transferrin saturation; ND, not determined; BM iron, bone marrow iron staining; +, positive bone marrow iron staining.

**Table 2:** Hemoglobin response (hemoglobin increase >20 g/L or to ≥120 g/L; in the 2010 Auerbach trial target Hb ≥110 g/L), EOTP ferritin, and EOTP TSAT for the seven published RCTs comparing ESA ± iron supplementation.

Trial	Hemoglobin response (%)	EOTP ferritin (*μ*g/L)	EOTP TSAT (%)
Auerbach 2004 [26]	25/36/68/68^a^	NR	NR
Auerbach 2010 [31]	72/82^b^	50/539^d^	−0.4/6.7^d^
Bastit et al. [29]	73/86^b^	NR	NR
Hedenus et al. [28]	53/93^b^	112/400^b^	20/30^b^
Henry et al. [27]	41/45/73^c^	−96/−14/344^e^	−14/−2.7/−1.8^e^
Pedrazzoli et al. [30]	70/93^b^	NR	NR
Steensma et al. [32]	65/67/70^c^	372/426/726^c^	24/28/24^c^

^a^control/p.o. iron/bolus doses iron/total dose infusion iron; ^b^control/i.v. iron; ^c^control/p.o. iron/i.v. iron; ^d^mean change control/i.v. iron; ^e^mean change control/p.o. iron/i.v. iron. Abbreviations: EOTP, end of treatment period; TSAT, transferrin saturation; NR, not reported; i.v., intravenous.
